# Neural Correlates of Uncertainty Processing During Decision-Making in Individuals with Different Levels of Worry Proneness

**DOI:** 10.3390/bs16091518

**Published:** 2026-08-28

**Authors:** Xueli Cai, Mengxue Wu, Hong Tang, Rui Yang, Ju Fan

**Affiliations:** Psychological Research and Counseling Center, Southwest Jiaotong University, Chengdu 611756, China

**Keywords:** worry proneness, ambiguity, risk, uncertainty, fMRI

## Abstract

Background: Worry proneness has been proposed to influence uncertainty processing; however, its neural mechanisms during different forms of uncertain decision-making remain poorly understood. This study used functional magnetic resonance imaging (fMRI) to investigate behavioral and neural responses associated with risky and ambiguous decision-making and to examine how individual differences in worry proneness relate to uncertainty-related neural processing. Methods: Participants completed a modified ambiguity–risk decision-making task during fMRI scanning. Whole-brain, region-of-interest (ROI), and continuous PSWQ-based correlation analyses were conducted to characterize neural responses associated with uncertainty type and individual differences in worry proneness. Results: Behavioral analyses revealed no significant effects of worry proneness or uncertainty type on gambling choices or reaction times. Whole-brain analyses showed that risky decisions elicited greater activation than ambiguous decisions in the right insula, right inferior parietal lobule, and left supramarginal gyrus, whereas ambiguous decisions preferentially recruited the right superior temporal gyrus. An exploratory peak-level interaction between worry proneness and uncertainty type was identified in the right middle frontal gyrus (BA6), with higher worry proneness associated with lower BA6 activation during risky decision-making. Continuous analyses further showed that BA6 activation during risky decisions was negatively correlated with PSWQ scores. ROI analyses demonstrated greater activation during risky than ambiguous decisions in the bilateral caudate nucleus, left insula, and right inferior frontal gyrus. Conclusions: These findings demonstrate that risky and ambiguous decision-making are associated with partially dissociable patterns of neural activation. Worry proneness was not associated with widespread alterations in behavioral performance or uncertainty-related neural responses; however, exploratory analyses identified localized associations between worry proneness and prefrontal neural responses during risky decision-making. These findings suggest that individual differences in worry proneness may be related to selective variation in specific neural processes rather than broad alterations in the neural systems supporting uncertainty processing.

## 1. Introduction

In real-world contexts, individuals are frequently required to make decisions under conditions of incomplete information, a situation commonly referred to as decision-making under uncertainty. Traditional economic theory distinguishes uncertainty into two qualitatively different forms: risk and ambiguity. Risk refers to situations in which the probability distribution of outcomes is known to the decision maker, whereas ambiguity describes contexts in which probabilistic information is unavailable or ill-defined (Knight, 1921). This conceptual distinction has been widely adopted in cognitive neuroscience to elucidate the neural mechanisms underlying different forms of uncertainty processing (Rushworth & Behrens, 2008; Ambrase et al., 2024; Timashkov et al., 2025). Accordingly, differentiating risk from ambiguity provides an important framework for understanding the cognitive and neural architecture of uncertainty processing during decision formation.

In decision-making tasks, risk and ambiguity are associated with distinct behavioral tendencies and partially dissociable neural mechanisms. For example, studies directly comparing risk and ambiguity conditions have consistently shown that ambiguous options are more likely to elicit avoidance behavior, a phenomenon known as ambiguity aversion, which represents a classic finding in decision theory (Ellsberg, 1961). Functional magnetic resonance imaging (fMRI) studies further indicate that risk and ambiguity engage partially dissociable neural systems. Limbic and prefrontal regions are sensitive to ambiguity level and subjective value, whereas striatal regions contribute to value computation and action selection during uncertain choice (Levy et al., 2010; Poudel et al., 2020; Pushkarskaya et al., 2015). Consistent with this view, Hsu et al. (2005) reported that striatal activation was reduced under ambiguity relative to risk, whereas ambiguity-related uncertainty was associated with increased engagement of the amygdala and orbitofrontal cortex. More recent meta-analytic evidence has further confirmed that uncertainty processing is supported by partially overlapping yet functionally dissociable neural networks involving the striatum, insula, prefrontal cortex, and limbic regions (Feng et al., 2022; Timashkov et al., 2025). Together, these findings suggest that risk and ambiguity engage partially distinct neural systems and provide a basis for investigating how individual differences in worry proneness are associated with neural responses to different forms of uncertainty.

Worry proneness is a dispositional tendency toward repetitive, future-oriented negative thinking in response to uncertainty and is closely associated with intolerance of uncertainty (Dugas et al., 1998). As a core cognitive characteristic of generalized anxiety disorder and a marker of anxiety vulnerability (Battle, 2013; Cox et al., 2022; Newman et al., 2013), studying worry proneness in healthy individuals provides an approach for examining individual differences in uncertainty processing. Consistent with this view, theoretical and neuroimaging studies suggest that worry and related anxiety traits are associated with altered recruitment of prefrontal, insular, limbic, and striatal regions involved in uncertainty processing (Grupe & Nitschke, 2013; Timashkov et al., 2025). Altered neural responses have been reported during uncertain threat anticipation (Morriss et al., 2022; Geng et al., 2018), in patients with generalized anxiety disorder (Assaf et al., 2018), and in resting-state functional connectivity among individuals with elevated worry proneness (Zhang et al., 2023), suggesting that worry may influence neural systems supporting uncertainty processing.

Despite growing evidence that risk and ambiguity recruit partially dissociable neural systems (Hsu et al., 2005; Timashkov et al., 2025), it remains unclear whether individual differences in worry proneness modulate neural responses to these two forms of uncertainty during active decision-making. Previous neuroimaging studies have primarily focused on clinical anxiety (Assaf et al., 2018), resting-state functional organization (Zhang et al., 2023), or uncertain threat anticipation (Morriss et al., 2022; Geng et al., 2018), whereas considerably less is known about how worry proneness influences neural activity during risky and ambiguous decision-making. Because risky and ambiguous decisions involve distinct computational demands (Hsu et al., 2005; Poudel et al., 2020; Wu et al., 2021), examining worry proneness in this context may provide new insights into how individual differences shape neural responses to different forms of uncertainty.

The present study employed functional magnetic resonance imaging (fMRI) together with the Card-Deck task adapted from Hsu et al. (2005), which explicitly dissociates risky and ambiguous decisions by manipulating the availability of probabilistic information. Using this well-established task, we investigated the behavioral and neural mechanisms underlying risky and ambiguous decision-making and examined how individual differences in worry proneness are associated with uncertainty-related neural processing. Whole-brain analyses, complemented by hypothesis-driven region-of-interest (ROI) analyses and continuous PSWQ-based correlation analyses, were performed to characterize neural responses associated with uncertainty type and individual differences in worry proneness.

Based on previous neuroimaging findings, we hypothesized that risky and ambiguous decisions would be associated with partially dissociable patterns of neural activation during uncertainty processing. Specifically, because risky decisions provide explicit probability information, we expected greater recruitment of regions involved in probabilistic evaluation and goal-directed decision-making, particularly striatal regions implicated in previous studies (Hsu et al., 2005; Levy et al., 2010; Poudel et al., 2020; Timashkov et al., 2025). In contrast, because ambiguous decisions lack explicit probability information and require decisions under incomplete knowledge, we expected greater involvement of regions associated with affective evaluation and uncertainty representation (Hsu et al., 2005; Pushkarskaya et al., 2015).

Furthermore, given that worry proneness is characterized by heightened sensitivity to uncertainty and intolerance of uncertain situations (Dugas et al., 1998; Grupe & Nitschke, 2013), we hypothesized that individual differences in worry proneness would be associated with variability in neural responses during risky and ambiguous decision-making. Specifically, we expected worry proneness to be associated with differential recruitment of neural systems involved in affective and cognitive evaluation of uncertain outcomes.

## 2. Methods

### 2.1. Participants

A total of 89 college students were selected from an initial screening sample of 1,224 students who completed the Penn State Worry Questionnaire (PSWQ; Zhong et al., 2009). Participants were recruited using an extreme-group design, with individuals scoring within the top 10% of the PSWQ distribution classified as the high-worry-proneness (HWP) group and those scoring within the bottom 10% classified as the low-worry-proneness (LWP) group.

Before the final analyses, nine participants were excluded. Specifically, six participants were excluded because their fMRI data were unusable owing to excessive head motion, poor task performance, or data loss during image acquisition. Three additional participants were excluded because incomplete behavioral data prevented reliable confirmation of group assignment. Thus, the final analytical sample comprised 80 participants, including 41 individuals in the HWP group and 39 individuals in the LWP group. All participants completed the Self-Rating Anxiety Scale (SAS) and the Self-Rating Depression Scale (SDS) (Zung, 1965, 1971). Although participants were recruited using an extreme-group design to maximize between-group contrasts, PSWQ scores were additionally analyzed as a continuous measure in subsequent correlation analyses.

All participants met the MRI eligibility criteria (i.e., no metallic implants, no claustrophobia, and no contraindications for MRI scanning). None had a history of neurological or psychiatric disorders, and none were taking psychoactive medications at the time of testing. The study was approved by the Ethics Committee of the Clinical Hospital of Chengdu Brain Science Institute in accordance with the Declaration of Helsinki (IRB No. 2020036). Written informed consent was obtained from all participants prior to participation.

### 2.2. Materials and Tasks

The difference between risky and ambiguous uncertainty is illustrated by the Ellsberg paradox (Ellsberg, 1961). The present study employed a Card-Deck paradigm adapted from Hsu et al. (2005). In this Card-Deck paradigm, participants chose between accepting a guaranteed payoff or placing a bet on either a red or blue card. The risky deck: The total number of cards is known (e.g., 20 cards), and the respective quantities of red and blue cards are also known—for example, 10 red cards and 10 blue cards. The ambiguous deck: The total number of cards is known (e.g., 20 cards), but the composition of red and blue cards is completely unknown. There were three types of key-press choices in the experiment. Two of them involved placing a bet on one of the two options in a binary gamble (Keys 1 or 2). Such gambles involved uncertainty, as the outcome could be either a monetary gain or zero. The third option was to accept a fixed, guaranteed amount (Key 3). Participants who chose to gamble selected between the red and blue cards by pressing Keys 1 and 2, respectively; those who chose not to gamble accepted the certain amount by pressing Key 3.

No outcome feedback was provided on each trial. Upon completion of the experiment, one trial was randomly selected, and each participant’s payment was determined based on their choice in that trial. The number of cards and potential monetary outcomes varied across trials. The task consisted of a total of 48 trials presented within a single functional run, including 24 ambiguity trials and 24 risk trials. In each trial, a blank screen was first presented for a jittered interval of 2, 4, or 6 s, followed by the presentation of the Card-Deck stimulus for 13 s. Participants were instructed to make their choice within the 13 s by pressing the corresponding response key. Once a response was made, the Card-Deck stimulus disappeared immediately, and a blank screen was presented for the remaining time to maintain a constant trial duration of 13 s. The subsequent trial began immediately after the completion of this period. Trial order was pseudorandomized across conditions, and intertrial intervals were jittered to reduce temporal predictability. 

**Figure 1:**
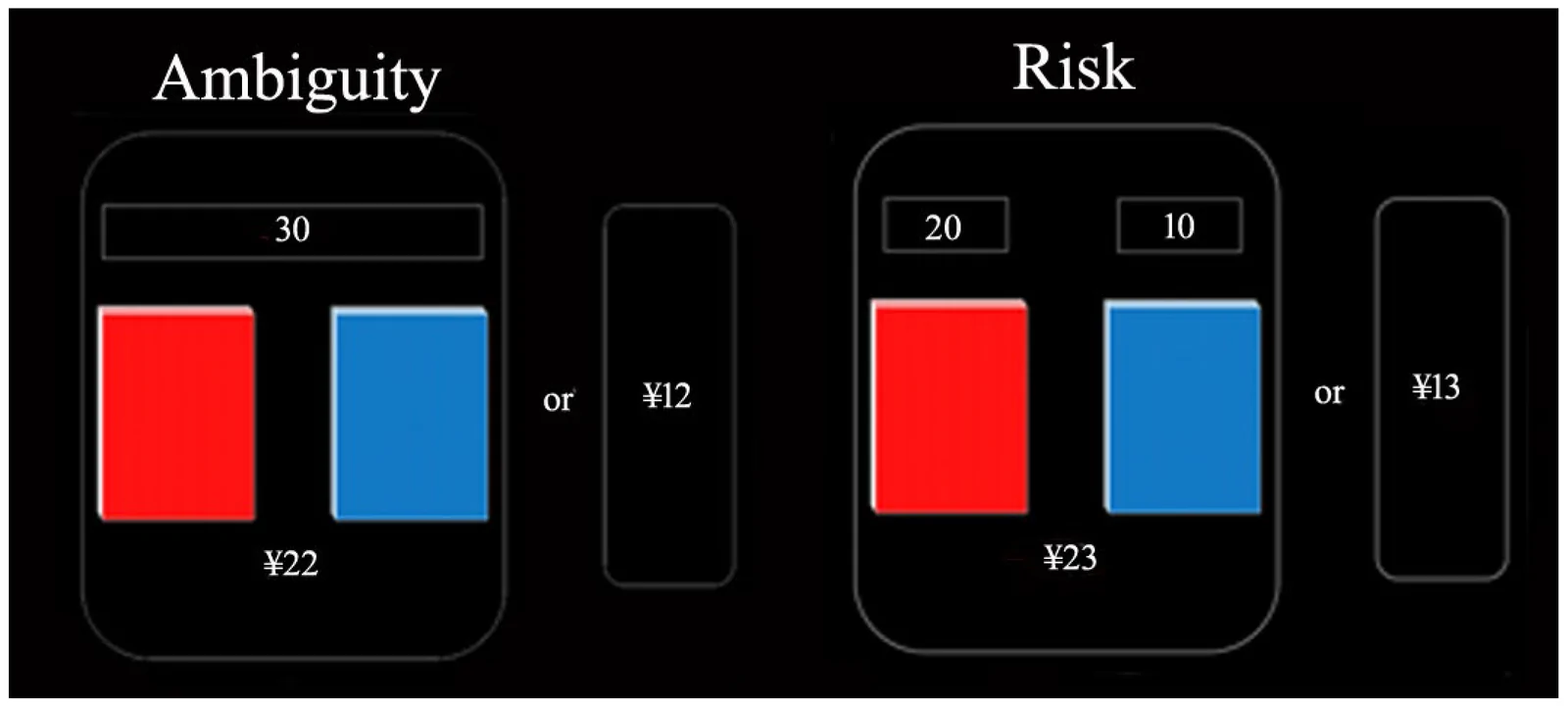
Card-deck task treatment. Ambiguity refers to unknown exact proportions, whereas risk refers to known card counts (indicated by numbers above each deck). Red and blue indicate the two types of cards in each deck; the colors themselves do not represent different experimental conditions or outcomes.

A representative trial is illustrated in Figure 1. In the ambiguity condition, there were a total of 30 cards, but the exact number of red and blue cards was unknown. If a participant chose to gamble and the color of their selected card matched the computer’s random selection, they received ¥22; if the colors did not match, they received ¥0. Alternatively, if they chose the fixed amount, they received ¥12. In the risk condition, there were 20 red cards and 10 blue cards. If the color of the participant’s chosen card matched the computer’s random selection, they received ¥23; if the colors differed, they received ¥0. The fixed amount in this condition was ¥13.

### 2.3. Behavioral Data Analysis

Behavioral measures were analyzed using a 2 (Worry Proneness: high vs. low) × 2 (Uncertainty Type: ambiguity vs. risk) mixed experimental design, with worry proneness as a between-subjects factor and uncertainty type as a within-subjects factor. The proportion of gambling choices and mean reaction times (RTs) served as the primary dependent variables. 

### 2.4. fMRI Data Acquisition

Functional magnetic resonance imaging (fMRI) data were acquired using a Siemens TRIO 3.0 T full-body MRI scanner (Siemens, Erlangen, Germany). Anatomical images were acquired using a three-dimensional T1-weighted (3DT1) sequence. The acquisition parameters were as follows: matrix size = 256 × 256, 156 sagittal slices, voxel size = 1 × 1 × 1 mm^3^, repetition time (TR) = 5.96 ms, echo time (TE) = 1.964 ms, inversion time (TI) = 450 ms, and flip angle = 12°.

Functional images were acquired using a gradient-echo echo-planar imaging (EPI) sequence with prospective acquisition correction (PACE). The acquisition parameters were as follows: repetition time (TR) = 2000 ms, echo time (TE) = 30 ms, flip angle = 90°, field of view (FOV) = 220 mm, acquisition matrix = 64 × 64, and 32 axial slices. The voxel size was 3.44 × 3.44 × 3.2 mm^3^. Images were acquired parallel to the anterior commissure–posterior commissure (AC–PC) plane. A total of 419 functional volumes were acquired for each participant, with an acquisition duration of approximately 14 min.

### 2.5. fMRI Data Analyses

Functional images were preprocessed and analyzed using SPM12 (Wellcome Department of Imaging Neuroscience, London, UK; https://www.fil.ion.ucl.ac.uk/spm/ (accessed on 24 August 2026)) (Friston et al., 1997). Preprocessing included slice-timing correction followed by rigid-body realignment to correct for head motion and estimate six motion parameters (three translations and three rotations). The first two functional volumes were discarded to allow for magnetization equilibrium. Functional images were then normalized to the Montreal Neurological Institute (MNI) template with a voxel size of 3 × 3 × 3 mm^3^ and spatially smoothed using a 6-mm full-width at half-maximum (FWHM) Gaussian kernel.

During MRI acquisition, participants were instructed to minimize head movement, and they were reminded to remain still when noticeable movement occurred. Head motion was assessed using the six rigid-body realignment parameters obtained during preprocessing. Participants showing head motion exceeding 3 mm translation or 3° rotation were excluded. One participant was excluded due to excessive head motion. For the remaining participants, framewise displacement (FD) was calculated based on the six rigid-body motion parameters to further quantify temporal head-motion fluctuations. Mean FD, maximum FD, and the number of volumes exceeding an FD threshold of 0.5 mm were compared between the high- and low-worry groups. No volume censoring (scrubbing) was performed because no participant exceeded the predefined FD criterion requiring removal of high-motion volumes (FD > 0.5 mm). No significant group differences were observed in any FD-related measure (all *p*s > 0.05; Supplementary Table S1), indicating comparable head-motion levels between groups.

Following preprocessing, task-related neural responses were modeled using the general linear model (GLM). Two regressors corresponding to ambiguous and risky decision trials were specified and convolved with the canonical hemodynamic response function. The duration of each decision event was modeled using the participant-specific reaction time, reflecting the period of active decision formation. The six realignment parameters were included as nuisance regressors to account for residual motion-related variance. For each participant, first-level contrast images for the ambiguity and risk conditions were generated and entered into second-level random-effects analyses.

The first-level contrast images were entered into a second-level flexible factorial model implemented in SPM12 to examine the main effects of worry proneness group (high vs. low), uncertainty type (ambiguity vs. risk), and their interaction. Uncertainty type was modeled as a within-subject factor, worry proneness group as a between-subject factor, and participant as a random factor to account for repeated measurements. Whole-brain statistical inference was performed using voxel-wise family-wise error (FWE) correction at *p* < 0.05. A minimum cluster extent threshold of k ≥ 10 voxels was applied for reporting significant clusters.

To further characterize the whole-brain interaction effect, parameter estimates were extracted from the identified interaction cluster. These extracted values were used for visualization of the interaction pattern and exploratory correlation analysis with PSWQ scores. Specifically, Pearson correlation analysis was conducted between PSWQ scores and activation estimates extracted from the interaction cluster to examine whether individual differences in worry proneness were associated with localized neural responses. FDR correction was applied to this exploratory correlation analysis.

To further investigate the neural mechanisms underlying uncertainty-related decision-making, region-of-interest (ROI) analyses were conducted based on previous neuroimaging findings and the ALE meta-analysis reported by Timashkov et al. (2025). Six-millimeter-radius spherical ROIs were defined for the bilateral caudate nucleus (left: −10, 8, 0; right: 10, 6, 2), bilateral anterior insula (BA13; left: −30, 22, 4; right: 30, 20, 8), left dorsal anterior cingulate cortex (dACC; BA32; −4, 8, 46), and right inferior frontal gyrus (BA9; 44, 8, 24), all of which were identified in the meta-analysis. Because worry proneness is closely associated with affective processing, bilateral amygdala ROIs (left: −22, −4, −18; right: 24, −2, −18) were additionally included as hypothesis-driven regions based on previous evidence implicating the amygdala in anxiety-related emotional processing (Grupe & Nitschke, 2013). Mean parameter estimates (beta values) for the ambiguity and risk conditions were extracted from the first-level GLM using MarsBaR (version 0.42).

For each ROI, extracted beta values were analyzed using a 2 (worry proneness group: high vs. low) × 2 (uncertainty type: ambiguity vs. risk) mixed-design ANOVA. When significant main effects or interactions were observed, Bonferroni-adjusted pairwise comparisons were performed to examine simple effects.

To further examine continuous associations between worry proneness and ROI neural responses, Pearson correlation analyses were conducted between PSWQ scores and activation estimates extracted from predefined ROIs under ambiguity and risk conditions. FDR correction was applied to ROI-based correlation analyses.

## 3. Results

### 3.1. Demographic Data

As shown in Table 1, the HWP and LWP groups did not differ significantly in gender distribution (χ^2^(1) = 0.179, *p* = 0.673). As expected, the HWP group scored significantly higher on the PSWQ than the LWP group (t(78) = 27.323, *p* < 0.001). There were no significant between-group differences in SAS or SDS scores (both *p*s > 0.05).

### 3.2. Behavioral Results

Across all participants and conditions, the proportion of gambling choices (M = 0.565, SD = 0.200) was significantly higher than the proportion of certain choices (M = 0.433, SD = 0.201), t(79) = 2.959, *p* < 0.01, indicating an overall tendency to select gambling options rather than certain payoffs.

To examine whether worry proneness and uncertainty type modulated choice behavior, a 2 (Worry proneness: high vs. low; between-subjects) × 2 (Uncertainty type: ambiguity vs. risk; within-subjects) mixed-design ANOVA was conducted on the proportion of gambling choices.

The analysis revealed no significant main effect of worry proneness, uncertainty type, or the interaction effect between them (all *p*s > 0.05). These findings indicate that gambling preferences did not differ systematically across worry proneness groups or uncertainty conditions.

Reaction Time (RT)

To examine overall response speed during decision-making, mean reaction times (RTs) were analyzed using a 2 (worry proneness: high vs. low; between-subjects) × 2 (uncertainty type: ambiguity vs. risk; within-subjects) mixed-design ANOVA, with RTs averaged across decision choices. The analysis revealed no significant main effect of worry proneness, no significant main effect of uncertainty type, and no significant interaction between worry proneness and uncertainty type (all *p*s > 0.05).

### 3.3. Imaging Results

#### 3.3.1. Whole-Brain Results

**Figure 2:**
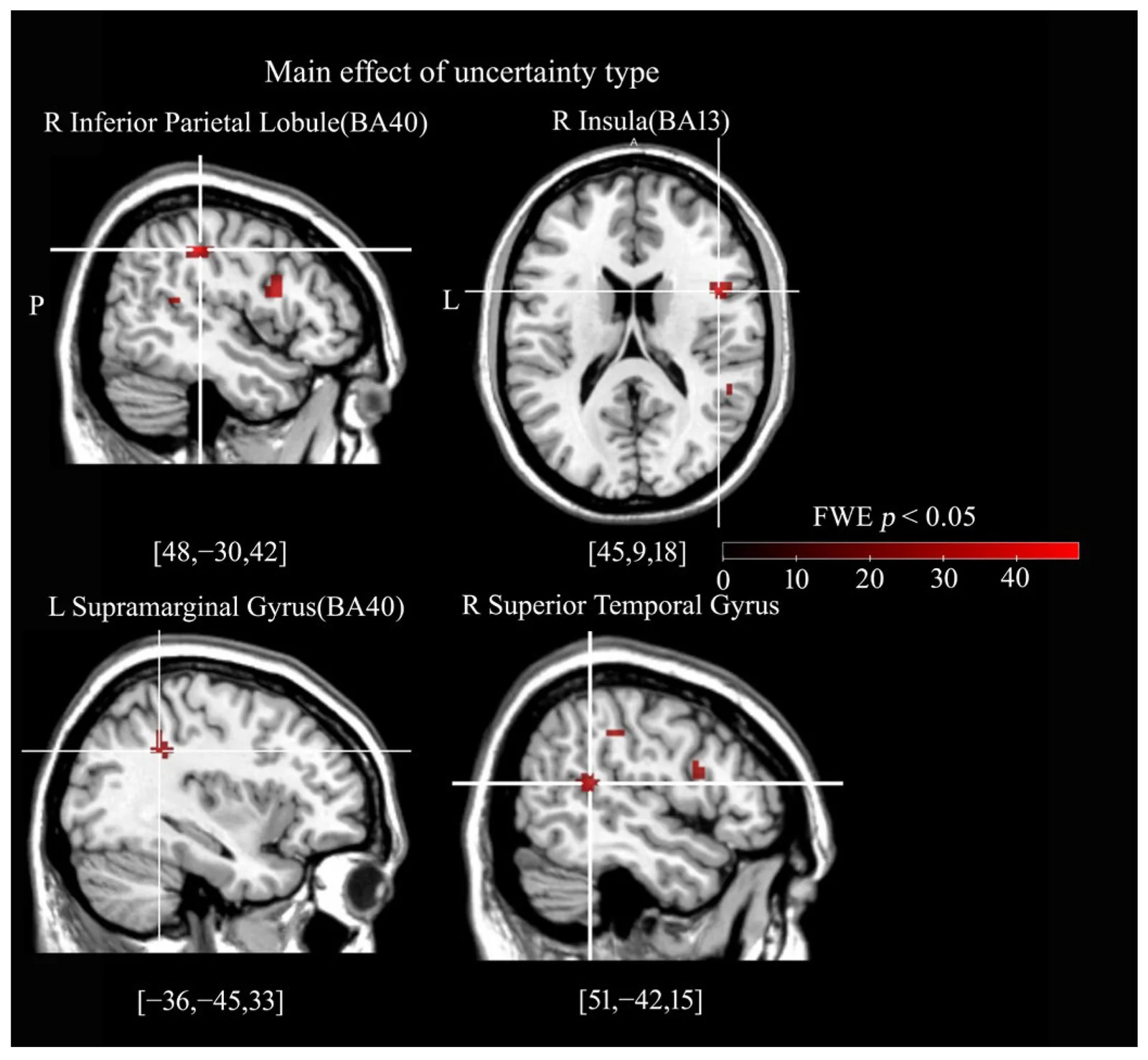
Whole-brain main effect of uncertainty type revealed by the 2 × 2 mixed-design ANOVA (worry proneness group × uncertainty type). L = left; R = right.

As shown in Table 2 and Figure 2, whole-brain analysis revealed a significant main effect of uncertainty type in the right inferior parietal lobule, right insula, left supramarginal gyrus, and right superior temporal gyrus. Post hoc comparisons revealed that the risky condition elicited greater activation than the ambiguous condition in the right insula, right inferior parietal lobule, and left supramarginal gyrus. By contrast, the ambiguous condition evoked greater activation than the risky condition in the right superior temporal gyrus.

**Figure 3:**
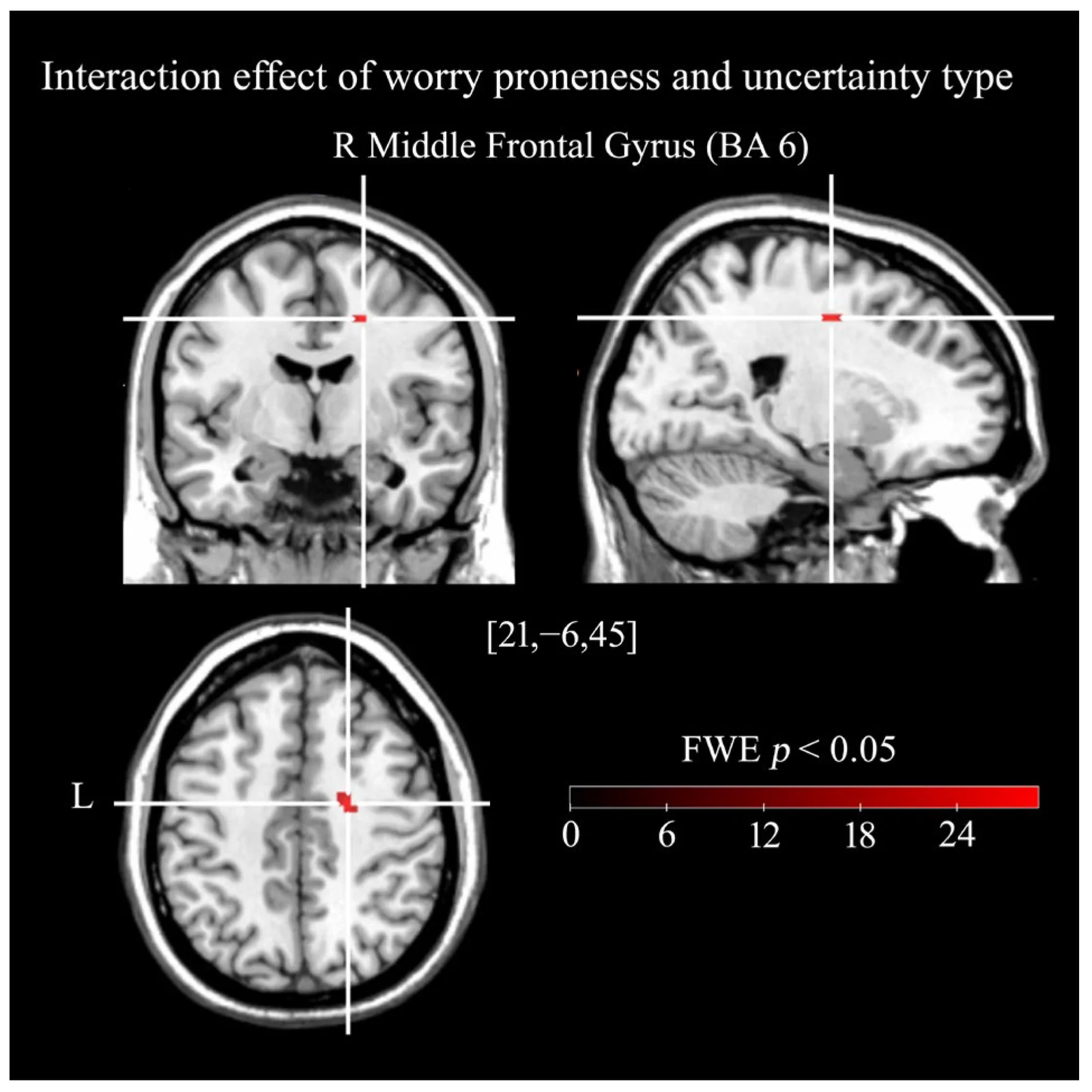
Exploratory whole-brain interaction between worry proneness group and uncertainty type in the right middle frontal gyrus (BA6). L = left; R = right.

No interaction cluster survived the predefined cluster-extent threshold of k ≥ 10. However, a small cluster in the right middle frontal gyrus (BA6) showed a peak-level FWE-corrected interaction effect, although its spatial extent did not meet the predefined extent criterion (k = 6) (Figure 3). Given the limited cluster size, this finding was considered exploratory. Parameter estimates were extracted from this cluster for follow-up analyses. 

Treating worry proneness as a continuous individual-difference variable, parameter estimates extracted from the right middle frontal gyrus (BA6) peak were correlated with PSWQ scores. A significant negative association was observed during risky decision-making (r = −0.33, *p* = 0.003, FDR-adjusted *p* = 0.006), whereas no significant association was found during ambiguous decision-making (r = −0.106, *p* = 0.351, FDR-adjusted *p* = 0.351). Exploratory simple-effect analyses further showed that high-worry individuals (M = 0.07) exhibited lower activation in the right middle frontal gyrus than low-worry individuals (M = 0.25) during risky decisions, t(78) = −3.13, *p* = 0.002, whereas no significant group difference was observed during ambiguous decisions.

#### 3.3.2. ROI Results

ROI analyses were conducted on the bilateral caudate nucleus, bilateral insula, bilateral amygdala, left dorsal anterior cingulate cortex (dACC), and right inferior frontal gyrus (IFG). Extracted beta values were analyzed using 2 × 2 mixed-design ANOVAs, with worry proneness group (high vs. low) as a between-subject factor and uncertainty type (ambiguity vs. risk) as a within-subject factor. All ROIs were predefined based on previous neuroimaging studies and meta-analytic findings. Bonferroni correction was applied for post hoc pairwise comparisons, and FDR correction was applied to ROI-based correlation analyses.

Caudate nucleus

For the bilateral caudate nucleus, a significant main effect of uncertainty type was observed in the left caudate nucleus, F(1,78) = 6.065, *p* = 0.016, ηp^2^ = 0.072. Activation was lower during ambiguous decisions (M = 0.281) than during risky decisions (M = 0.382). Similarly, the right caudate nucleus showed a significant main effect of uncertainty type, F(1,78) = 7.938, *p* = 0.006, ηp^2^ = 0.092, with lower activation during ambiguous decisions (M = 0.358) compared with risky decisions (M = 0.479). Neither the main effect of worry proneness nor the worry proneness × uncertainty type interaction was significant in either caudate nucleus (all *p*s > 0.05).

Insula

For the bilateral insula, the left insula showed a significant main effect of uncertainty type, F(1,78) = 5.196, *p* = 0.025, ηp^2^ = 0.062, whereas the corresponding effect in the right insula was not significant. Post hoc pairwise comparisons indicated that left insula activation was lower during ambiguous decisions (M = 0.497) than during risky decisions (M = 0.586). Neither the main effect of worry proneness nor the worry proneness × uncertainty type interaction was significant in either insula (all *p*s > 0.05).

Inferior frontal gyrus

The right IFG (BA9) exhibited a significant main effect of uncertainty type, F(1,78) = 12.168, *p* = 0.001, ηp^2^ = 0.135. Post hoc pairwise comparisons showed that activation was lower during ambiguous decisions (M = 0.749) than during risky decisions (M = 0.968). Neither the main effect of worry proneness nor the interaction between worry proneness and uncertainty type was significant (all *p*s > 0.05).

Amygdala

For the bilateral amygdala, exploratory ROI analysis revealed a nominal worry proneness × uncertainty type interaction in the left amygdala, F(1,78) = 4.537, *p* = 0.036, ηp^2^ = 0.055. Follow-up simple effect analyses were conducted to characterize this interaction pattern. Among individuals with HWP, left amygdala activation was higher during ambiguous decisions (M = 0.168) than during risky decisions (M = 0.094), t(40) = 2.017, *p* = 0.05, Cohen’s dz = 0.32. In contrast, no significant difference between ambiguity (M = 0.134) and risk conditions (M = 0.199) was observed in individuals with LWP (*p* > 0.05). Neither the main effect of uncertainty type nor the main effect of worry proneness was significant in the left amygdala. For the right amygdala, neither the main effect of uncertainty type, the main effect of worry proneness, nor the interaction effect reached significance (all *p*s > 0.05). This exploratory finding may suggest a potential difference in amygdala responses associated with worry proneness; however, it should be interpreted cautiously.

Dorsal anterior cingulate cortex

For the left dACC(BA32), neither the main effect of uncertainty type, the main effect of worry proneness, nor their interaction reached significance (all *p*s > 0.05).

Finally, continuous PSWQ-based correlation analyses were conducted to examine associations between worry proneness and ROI activation. None of the correlations between PSWQ scores and activation estimates extracted from predefined ROIs survived FDR correction for multiple comparisons (all adjusted *p*s > 0.05).

## 4. Discussion

The present study investigated the behavioral and neural mechanisms underlying decision-making under risk and ambiguity and examined how individual differences in worry proneness are associated with uncertainty-related processing. Overall, the findings indicate that risk and ambiguity are associated with partially distinct patterns of neural activation, whereas worry proneness was associated with limited and localized differences in uncertainty-related neural responses. Behaviorally, participants generally preferred gambling over certain options, but neither gambling choices nor response times varied as a function of worry proneness or uncertainty type. At the neural level, whole-brain analyses revealed greater activation in insular and parietal regions during risky decisions relative to ambiguous decisions, while ROI analyses further showed similar uncertainty-related modulation in the bilateral caudate nucleus and right inferior frontal gyrus. Although worry proneness was not associated with widespread alterations in uncertainty-related neural activity, exploratory analyses revealed localized differences, including a peak-level whole-brain interaction in the right middle frontal gyrus (BA6) during risky decisions and a nominal ROI interaction in the left amygdala. Together, these findings suggest that individual differences in worry proneness are associated with specific neural responses during uncertainty processing rather than producing widespread alterations in uncertainty-related brain activity.

### 4.1. Distinct Neural Responses to Risk and Ambiguity

The present findings provide converging evidence that risk and ambiguity are associated with partially dissociable neural responses. This pattern is broadly consistent with recent coordinate-based meta-analyses demonstrating that different forms of uncertainty recruit partially overlapping yet functionally distinct neural networks (Feng et al., 2022; Timashkov et al., 2025). 

The anterior insula has been widely implicated in salience detection and the integration of interoceptive, emotional, and cognitive information (Menon & Uddin, 2010; Grupe & Nitschke, 2013). In addition, computational neuroimaging studies have shown that the anterior insula encodes risk prediction and risk prediction errors when outcome probabilities are explicitly available (Preuschoff et al., 2008). Accordingly, greater insular activation during risky decisions may reflect increased involvement in evaluating behaviorally relevant uncertainty when explicit probability information is available.

ROI analyses further revealed greater activation of the bilateral caudate nucleus during risky than ambiguous decisions, whereas worry proneness showed neither a main effect nor an interaction with uncertainty type. As a core component of the dorsal striatum, the caudate has been consistently implicated in goal-directed behavior, action–outcome learning, and the evaluation of action consequences (Balleine & O’Doherty, 2010; Delgado, 2007). Unlike ambiguity, risky decisions provide explicit probability information, allowing individuals to estimate potential outcomes more precisely and establish stable action–outcome expectations (Hsu et al., 2005). Such probabilistic computations are thought to rely on striatal mechanisms supporting probability evaluation, reward prediction, and risk computation (Berns & Bell, 2012). Notably, Poudel et al. (2020) identified convergent activation of the bilateral caudate during risky decision-making and greater left caudate convergence in the direct risk-versus-ambiguity contrast, whereas Wu et al. (2021) further demonstrated preferential recruitment of ventral striatal and putaminal regions during risk processing. Consistent with these findings, the present study likewise demonstrated greater bilateral caudate activation during risky than ambiguous decisions. The present findings therefore suggest that greater caudate recruitment during risk may reflect more structured evaluation of choice–outcome contingencies when the likelihoods of potential outcomes are explicitly available.

Whole-brain analyses revealed greater activation of the right inferior parietal lobule and left supramarginal gyrus during risky than ambiguous decisions. Although recent meta-analytic evidence has associated parietal recruitment more consistently with ambiguity processing (Feng et al., 2022; Wu et al., 2021), parietal regions are not specific to ambiguity and have been implicated in probability-based decision variables, action selection, and the integration of decision-relevant information (Platt & Glimcher, 1999; Huettel, 2006; Volz et al., 2003). In the present task, risky decisions provided explicit probability information that had to be integrated with potential reward magnitude and compared with a certain alternative. Greater IPL and supramarginal activation may therefore reflect increased demands on the selection, maintenance, and integration of multiple explicitly specified decision variables. Differences from previous meta-analytic findings may reflect heterogeneity in task structure, decision stage, and the contrasts used to isolate risk and ambiguity across studies (Wu et al., 2021).

Together with previous meta-analytic findings (Feng et al., 2022; Timashkov et al., 2025), the present results support the view that different forms of uncertainty engage partially distinct patterns of neural activity depending on the availability of probabilistic information.

### 4.2. Limited Influence of Worry Proneness on Uncertainty-Related Processing

Although worry proneness did not broadly alter neural responses during uncertainty-related decision-making, whole-brain analysis revealed an exploratory peak-level interaction at the right middle frontal gyrus (BA6). The right middle frontal gyrus (BA6) showed a localized association with worry proneness during risky decision-making. Specifically, individuals with higher worry proneness exhibited lower BA6 activation during risky relative to ambiguous decisions, and BA6 activity during risky decision-making was negatively correlated with continuous PSWQ scores. The BA6 region, which encompasses the dorsal premotor cortex, has been implicated in goal-directed action selection and the transformation of decision-related information into behavioral responses (Cisek & Kalaska, 2010; Nakayama et al., 2022; Tecilla et al., 2022). One possible interpretation is that higher worry proneness may be associated with altered recruitment of goal-directed control processes when explicit probability information is available. Although the peak voxel survived whole-brain FWE correction, the corresponding cluster did not meet the predefined extent threshold (k ≥ 10). Therefore, this finding should be interpreted cautiously and requires replication in larger independent samples.

Exploratory ROI analyses additionally revealed a nominal worry proneness × uncertainty type interaction in the left amygdala. Given the established role of the amygdala in affective evaluation and uncertainty-related threat processing (Hsu et al., 2005; Grupe & Nitschke, 2013; Geng et al., 2018; Xiang et al., 2025), this finding may suggest that higher worry proneness may be associated with differences in affective responses when probabilistic information is unavailable. However, because this effect was observed only in an exploratory ROI analysis and was not supported by corresponding whole-brain or behavioral findings, it should be considered preliminary and requires further replication.

### 4.3. Limitations

Several limitations should be acknowledged. First, although the present study employed a Card-Deck task adapted from Hsu et al. (2005) to dissociate risky and ambiguous decision-making, the relatively limited number of trials and the simplified laboratory paradigm may have reduced statistical power and ecological validity. Future studies incorporating larger numbers of trials and more naturalistic decision-making paradigms may help improve the robustness and generalizability of these findings.

Second, although continuous PSWQ-based correlation analyses were additionally conducted, the initial recruitment strategy relied on an extreme-group design to maximize contrasts between individuals with relatively high and low levels of worry proneness. This approach may limit the generalizability of the findings across the full continuum of worry proneness. Future studies using larger samples with dimensional sampling across the entire range of worry proneness are needed to further characterize the continuous relationship between worry proneness and neural responses during uncertainty processing.

Third, the present study recruited healthy individuals with varying levels of worry proneness rather than individuals with clinically diagnosed anxiety disorders. Therefore, caution is warranted when generalizing these findings to clinical populations characterized by pathological worry. Future studies including both nonclinical and clinical samples may help clarify whether the observed neural associations extend to anxiety-related psychopathology.

Finally, although the present study identified localized neural associations related to worry proneness, some worry-related findings, particularly the right middle frontal gyrus (BA6) interaction and the exploratory amygdala ROI effect, should be interpreted cautiously given their exploratory nature and require replication in larger independent samples.

## 5. Conclusions

The present study investigated the neural mechanisms underlying risky and ambiguous decision-making and examined the role of worry proneness in uncertainty-related processing. The present study demonstrates that risk and ambiguity are associated with partially dissociable neural responses, with risky decisions eliciting greater recruitment of bilateral caudate, insular, and inferior parietal regions. Worry proneness was not associated with widespread alterations in behavioral performance or uncertainty-related neural activity. However, continuous individual-difference analyses and exploratory neuroimaging findings identified localized associations between worry proneness and prefrontal responses during uncertain decision-making. These findings suggest that worry proneness may be related to selective variation in specific neural processes rather than broad alterations in the neural systems underlying uncertainty processing.

## Figures and Tables

**Table 1:** Participant fundamental information.

	HWP Group	LWP Group	*p*
Gender (Male/Female)	24/17	21/18	0.673
PSWQ score	62.02 ± 5.29	32.51 ± 4.29	<0.001
SAS score	35.40 ± 4.41	34.70 ± 3.66	0.460
SDS score	44.35 ± 4.11	45.13 ± 4.15	0.410

**Table 2:** Whole-brain results for the main effect of uncertainty type and the worry proneness × uncertainty type interaction (FWE-corrected, *p* < 0.05).

Main Effect of Uncertainty Type	BA	X	Y	Z	F	Voxels of Cluster (k)
Right Inferior Parietal Lobule (IPL)	40	48	−30	42	50.39	35
Right Sub-lobar, Insula	13	45	9	18	42.42	29
Left Supramarginal Gyrus (SMG)	40	−36	−45	33	30.66	20
Right Superior Temporal Gyrus (STG)	−	51	−42	15	36.82	10
**Interaction Effect of Worry Proneness and Uncertainty Type**						
Right Middle Frontal Gyrus (MFG)	6	21	−6	45	31.98	6

## Data Availability

The original contributions presented in this study are included in the article/Supplementary Material. Further inquiries can be directed to the corresponding author.

## Supplementary Materials

The following supporting information can be downloaded at: https://www.mdpi.com/article/10.3390/bs16091518/s1, Table S1: Comparison of head-motion parameters between high- and low-worry groups.
