# Supplementary material for: Neural Correlates of Uncertainty Processing During Decision-Making in Individuals with Different Levels of Worry Proneness
Source: Behav Sci (Basel). 2026 Aug 28;16(9):1518. doi: 10.3390/bs16091518 (PMC13603976; doi:10.3390/bs16091518)
Supplement: Supplementary file 1 [file behavsci-16-01518-s001.zip › behavsci-4417577-supplementary.pdf]

Supplementary Table S1. Comparison of head-motion parameters between high- and low-worry groups.

**Motion parameter**	**High worry proneness (n = 41)**	**Low worry proneness (n = 39)**	**t**	***p***
Mean framewise displacement (FD, mm)	0.081 ± 0.034	0.080 ± 0.040	0.18	0.858
Maximum FD (mm)	0.624 ± 0.898	0.715 ± 0.726	−0.50	0.62
Volumes with FD > 0.5 mm	2.44 ± 6.02	3.32 ± 7.05	−0.61	0.543
